# Supplementary material for: The effect of angiotensin receptor blockers on peripheral artery disease progression: A retrospective cohort study in Jordan
Source: PLoS One. 2025 Sep 11;20(9):e0330522. doi: 10.1371/journal.pone.0330522 (PMC12425201; doi:10.1371/journal.pone.0330522)
Supplement: Attachment — Submitted filename: rebttual letter.docx [file pone.0330522.s004.docx]

**The effect of angiotensin receptor blockers on peripheral artery disease progression: A retrospective cohort study in Jordan**

**PONE-D-25-31876**

**PLOS ONE**

**Mahar Zaid Kasasbeh**

**July, 2025**

**Dear Editor-in-Chief,**

I would like to express my sincere gratitude for considering our manuscript entitled: “**The effect of angiotensin receptor blockers on peripheral artery disease progression: A retrospective cohort study in Jordan**” for publication in your esteemed and scientifically respected journal. I kindly ask you to consider our revised version for further evaluation, taking into account that all comments and recommendations raised by your kind editorial office and the respected reviewer have been addressed thoroughly, as detailed in this response.

Please find below a point-by-point response to the editorial and reviewer comments. The comments are highlighted in bold, followed by our respective responses. Kindly notice that the reported lines in where the changes were made is based on the manuscript with track changes

**Journal requirements:**

**2.Thank you for stating the following in the Acknowledgments Section of your manuscript: [The authors would like to acknowledge the Deanship of Research at Jordan University of Science and Technology for approving this research (approval number: 825/2022).] We note that you have provided funding information that is not currently declared in your Funding Statement. However, funding information should not appear in the Acknowledgments section or other areas of your manuscript. We will only publish funding information present in the Funding Statement section of the online submission form.**

Thank you for your feedback. We appreciate your concern regarding the potential confusion between our funding statement and the acknowledgment section. We would like to clarify that our research did not receive any funding. The acknowledgment of the Deanship of Research at our university was included solely to recognize their support in facilitating IRB approval and providing access to the hospital database.

**6. Please review your reference list to ensure that it is complete and correct. If you have cited papers that have been retracted, please include the rationale for doing so in the manuscript text, or remove these references and replace them with relevant current references. Any changes to the reference list should be mentioned in the rebuttal letter that accompanies your revised manuscript. If you need to cite a retracted article, indicate the article’s retracted status in the References list and also include a citation and full reference for the retraction notice.**

All references have been reviewed for retraction or corrections using the Retraction Watch database and PubMed. As of July, 31th, 2025, none of the cited works have been retracted or corrected in a way that affects the study’s content

**Additional Editor Comments to Author:**

**Reviewer #1 Comments to Author:**

**1. Consider citing methodological references or choosing more conventional sample size parameters.**

Thank you for the insightful comment. We have revised the sample size calculation section accordingly. Although we were unable to locate a recent and reliable reference for the equation previously used to estimate the odds ratio (OR) from the hazard ratio (HR), we have now provided a detailed explanation of the rationale behind our approach to the sample size calculation, along with its implications. Please refer to the revised sections in the Methods (lines 133–149) and Discussion (lines 231–233).

**2.The manuscript refers to 79.2% "lower limb amputation" without explicitly defining this as relative risk reduction. For clarity and transparency, consider rephrasing to “odds of limb amputation were 79.2% lower in the ARB group” and provide absolute event rates for both groups.**

We appreciate the suggestion. We have revised the sentence reporting the percentage of amputations in each group and have explicitly included the term absolute amputation rate to enhance clarity. Please see the updated text in the Results section (line 167).

**3. Provide a brief explanation of the Fontaine classification system in the methods for international readers.**

A brief explanation of the Fontaine classification system was already provided in the Methods section. Please refer to the Methods section (lines 110–113).

**4. The statement that ARBs were associated with "79.2% lower limb amputation" should be phrased more clearly.**

Thank you for the insightful comment. We appreciate your detailed recommendations. In response, we have revised the statement to read: “The odds of amputation in the ARBs group were 79.2% lower than in the control group.”

At the end, I would like to extend our sincere thanks for allowing us the opportunity to revise and resubmit our manuscript. We believe that the changes made in response to your kind guidance have substantially improved the manuscript, and we respectfully request your reconsideration for publication.

**Sincerely,**
On behalf of all authors
